# Seasonality and landscape characteristics impact species community structure and temporal dynamics of East African butterflies

**DOI:** 10.1038/s41598-021-94274-6

**Published:** 2021-07-23

**Authors:** Thomas Schmitt, Werner Ulrich, Andjela Delic, Mike Teucher, Jan Christian Habel

**Affiliations:** 1 Senckenberg German Entomological Institute, 15374 Müncheberg, Germany; 2 Zoology, Institute of Biology, Faculty Natural Sciences I, Martin Luther University Halle-Wittenberg, 06099 Halle (Saale), Germany; 3 Entomology and Biogeography, Institute of Biochemistry and Biology, Faculty of Science, University of Potsdam, 14476 Potsdam, Germany; 4 Department of Ecology and Biogeography, Nicolaus Copernicus University Toruń, 87-100 Toruń, Poland; 5 Evolutionary Zoology, Department of Biosciences, University of Salzburg, 5020 Salzburg, Austria; 6 Department of Geoecology, Institute of Geosciences and Geography, Martin Luther University Halle-Wittenberg, 06099 Halle (Saale), Germany

**Keywords:** Ecology, Evolution, Zoology, Ecology, Environmental sciences

## Abstract

Species community structures respond strongly to habitat changes. These are either driven by nature or human activities. The biota of East African drylands responds highly sensitively to natural and anthropogenic impacts. Thus, seasonality strongly influences resource availability in a cyclic manner during the year, with cyclic appearance of the different developmental stages of invertebrates, while man-made landscape transformations profoundly and permanently modify habitat structures and, as a consequence, species communities. Butterflies are an excellent model group for the study of the effects of seasonality, and to test for biodiversity responses to anthropogenic activities such as habitat modification, degradation and destruction. We performed transect counts of adult butterflies in riparian forests and their adjoining areas, either dry savannahs with occasional pasturing (i.e. near-natural status) or farmland areas with fields, gardens and settlements (i.e. highly degraded status with lack of original vegetation). Transects were set along the river beds as well as at 250 m and 500 m distances parallel to these rivers, with eight transects per distance class and site (i.e. 48 transects in total). We recorded habitat structures for each transect. Counts were conducted during the dry and the rainy season, with 16 repetitions for each single transect, i.e. eight per season and transect. We compiled trait data on morphology, geographic distribution, ecology, behaviour, and life-history for all butterfly species encountered. Our results show higher species richness and numbers of individuals in farmland transects compared with the savannah region. Seasonal fluctuations of the detectable species abundances between the rainy and dry season were severe. These fluctuations were much more pronounced for the savannah than the farmland area, i.e. was buffered by human activities. Farmland and savannah support two distinct butterfly communities, with generalist species being more common in the farmland communities. Strict habitat associations were comparatively weak and typical dry savannah and riparian forest species were not clearly restricted to the near natural landscape.

## Introduction

Various natural and anthropogenic factors drive ecosystems, and thus have important impacts on their species communities. Some of these factors are cyclic and temporary, others directed and permanent. A cyclic natural factor is seasonality, which strongly influences resource availability in ecosystems, with the consequence that it severely impacts the activity and occurrence of species, influences their developmental cycles, affects abundances, and thus community composition and structure over time^1^. Such seasonal community modifications are particularly pronounced for organisms with short generation cycles, such as most arthropods. For example, studies on butterfly imagoes in south-eastern Kenya’s coastal forests showed that their community structures and abundances differ markedly between the dry and rainy season^2^. The majority of anthropogenic activities impact ecosystems in a more permanent way, and subsequently also their species community structures, as revealed by various studies. Such anthropogenic activities often result in complete habitat destruction or at least strong modification of the habitat configuration, e.g. from interconnected into fragmented systems^3^. This frequently leads to a significant and permanent reduction of general habitat quality^4^, which severely impacts the occurrence of species and community assemblages^5^.

Species respond differently to the loss, fragmentation and degradation of habitats. Species with specialised habitat demands respond more sensitively to anthropogenic habitat transformations than species with a wide ecological amplitude^5^, while species requiring specific resources tolerate habitat modifications only to a rather limited extent^6^. In consequence, sedentary specialists suffer particularly under habitat destruction and subsequent habitat fragmentation^7^. Thus, changes in habitat conditions particularly impact those species with narrow adaptations to these particular environmental conditions^8^.

The drylands of East Africa belong to the tropical regions most strongly affected by extreme annual changes in the climatic conditions^9^, leading to remarkable fluctuations in species richness, community composition and abundance^10^. Until recently, this part of Africa was mainly covered by dry savannah, transgressed by temporarily water-carrying rivers. These rivers are naturally bordered by dense riparian vegetation, home of a unique flora and fauna with many specialised and endemic species^11^. The substantial increase of settlements and hence human activities (in particular subsistence agriculture) along these rivers and in general across the dryland areas has caused severe degradation of both the riparian forests^12,13^ and the dry savannahs^14^. The resilience of both ecosystems is rather limited, and many questions about the effects of human and natural impacts on these ecosystems and the species living therein remain unresolved.

**Figure 1:**
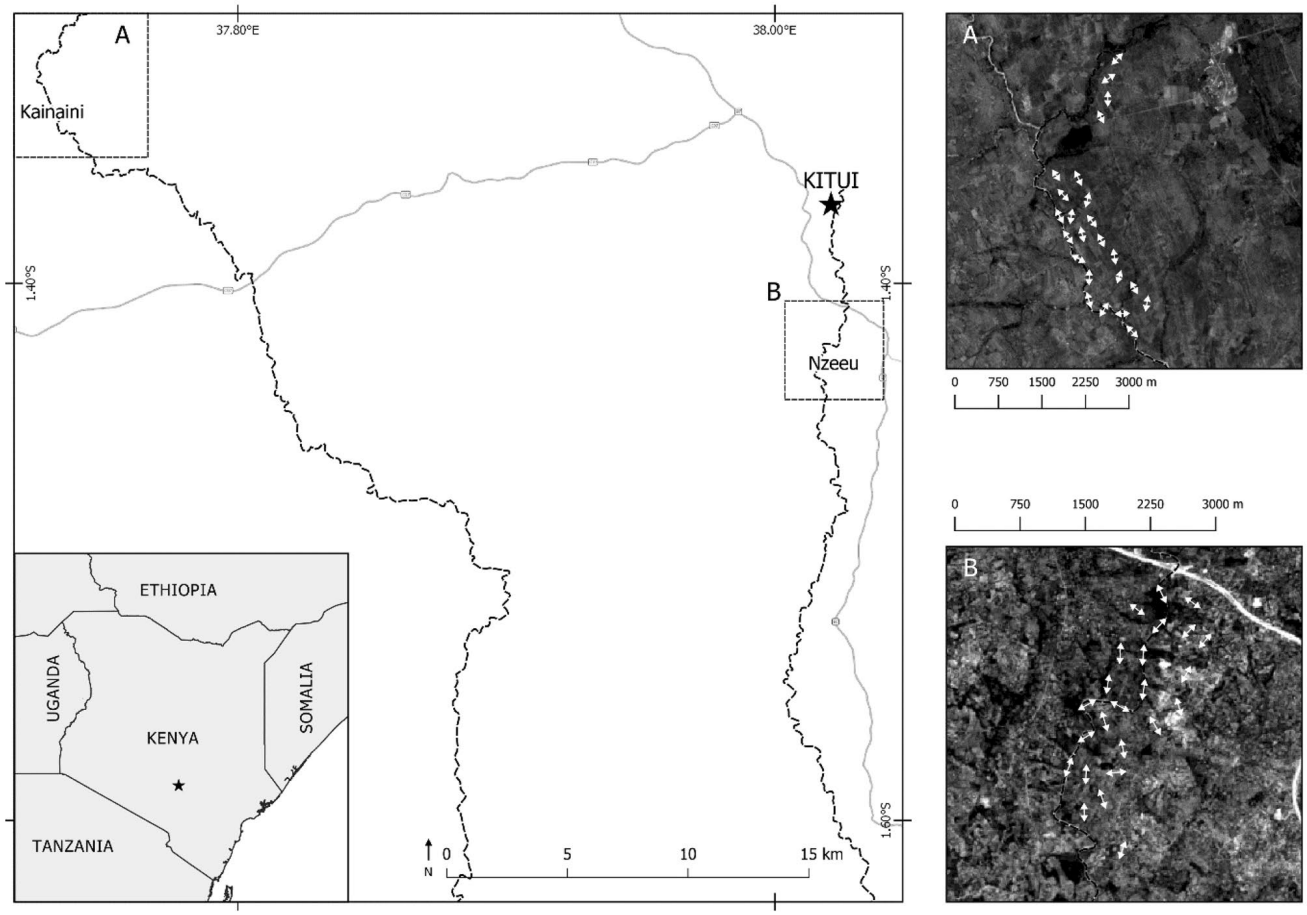
Location of our study region in Kenya (star in small inlaid map), enlarged map of our study region around Kitui, with the two study sites, Nzeeu River and Kainaini River, including all transects (aerial images).

Therefore, we analysed butterfly species communities along two rivers and in their adjoining dryland areas in southern Kenya. We established line-transects and performed standardized butterfly counts (modified after Pollard^15^). Transects were established along the banks of rivers and parallel to them at average distances of 250 m and 500 m, respectively. We established identical study designs in two landscape types in the vicinity of the city of Kitui in southern Kenya (Fig. 1): 1. In a densely populated and thus degraded landscape dominated by subsistence agriculture; and 2. In a still widely intact dry savannah with riparian forests along the river, only moderately affected by pasturing of live-stock. We recorded habitat structures for each transect, and counted butterflies along all transects during the dry and the rainy season. During each transect count, we recorded all individuals after their determination to species level. Morphological, distributional, ecological, behavioural, and life-history traits were assigned to each species encountered. Based on these data, we address the following research questions:

1. How do species richness, abundance and community structures differ in near natural habitats and in anthropogenic landscapes?
2. Are community structures in near natural habitats and in anthropogenic landscapes affected differentially by seasonal shifts?
3. How strongly do the riparian habitats influence the community structures in the adjoining areas?

## Results

**Figure 2:**
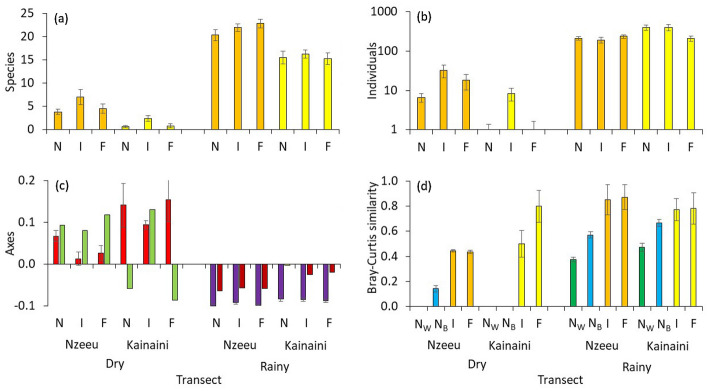
Species richness (**a**), and number of individuals (**b**), along the Nzeeu (orange) and Kainaini (yellow) rivers; given is the mean per transect. (**c**) Average scores of the dominant (explained variance: 91.5%; red: dry season, violet: rainy season) and subdominant (explained variance: 3.5%; green: dry season, brown: rainy season) PCoA eigenvectors across transects. (**d**) Bray–Curtis similarities of the intermediate (I) and far (F) transects to the near river transects (N), as well as average Bray–Curtis similarity within the eight near river transects N_W_ (green bars; only calculated for the rainy season; data insufficient for dry season) and the average similarities across the eight near river transects N_B_ (blue bars; data insufficient for Kainaini during the dry season). Error bars in (**a**), (**b**), and (**c**) denote standard errors from eight sample points in each transect. Error bars in (**d**) are based on 1000 bootstrap samples. Abbreviations: N—near river transects; I—transects at intermediate distance from river (i.e. 250 m); F—transects far from river (i.e. 500 m); N_W_—average Bray–Curtis similarity within the eight walks in the near river transects; N_B_—average similarities across the eight near river transects.

In total, we recorded 13,748 butterfly imagoes, representing 71 butterfly species along the transects of both study sites along two different river systems. Answering our first research question, we recorded more species in the agricultural site at Nzeeu River than in the transects established in the near native savannah environment at Kainaini River (Table 1, Fig. 2a). Irrespective of the differences in land-use in the two study sites, the dry season was significantly less rich in butterfly species on the wing compared to the rainy season (Tables 1, 2). Thus, totals of 35 and 53 species were found at Nzeeu during the dry and rainy seasons, respectively, i.e. an increase of 51%. The difference was more pronounced in the near-natural site at Kainaini with 11 and 51 species, respectively, i.e. an increase of 363% (Table 1, Fig. 2a). These differences in species numbers were also reflected by strong fluctuations in abundance of imagoes. At Nzeeu, we recorded 11.2 times more butterfly individuals during the rainy (5131) than during the dry season (459); this factor increased to 98.5 at Kainaini (8081 vs. 82; Table 1). Thus, comparing sites, the agricultural site at Nzeeu River had higher abundances during the dry season, the near-natural savannah site near Kainaini River during the rainy season. However, the latter was triggered by the very high abundance of one single butterfly species, i.e. *Belenois aurota* with 5805 individuals at Kainaini and 1028 at Nzeeu. Despite the more similar total abundances in the rainy season, we found significant differences in species richness between the Kainaini and Nzeeu river systems in direct comparisons of respective transects in both seasons, with the Kainaini transects always being less species-rich but only less rich in butterfly individuals during the dry season (Fig. 2a, b, Tables 1, 2).

**Table 1:** Basic data on the sample sizes (abundances), the total species richness S_total_, the average species richness per transect S_transect_, the number of species common to the three transect lines, and the respective β-diversity among transects for the four study site–season combinations.

Variable	Dry season		Rainy season	
	Nzeeu	Kainaini	Nzeeu	Kainaini
Abundance	459	82	5,131	8,081
S_total_	35	11	53	51
S_transect_	5.05 ± 0.14	1.25 ± 0.07	21.75 ± 0.19	15.67 ± 0.21
S_common_	9	4	32	24
β-diversity	0.85 ± 0.01	0.87 ± 0.02	0.59 ± 0.01	0.69 ± 0.01

Errors refer to bootstrapped standard errors of the mean.

**Table 2:** General linear modelling identified differences between study sites, transect distance to river bed, and season with respect to butterfly species richness and community composition (assessed by the dominant eigenvector of a principle component analysis of the species × transect matrix).

Variable	df	Species richness		Community composition	
		Partial η^2^	*P*	Partial η^2^	*P*
Study site	1	0.47	< 0.001	0.38	< 0.001
Distance to river	2	0.07	0.04	0.08	0.03
Season	1	0.70	< 0.001	0.31	< 0.001
Study site × distance to river	2	0.01	0.63	0.02	0.47
Study site × season	1	0.06	0.02	0.25	< 0.001
Distance to river × season	2	0.03	0.28	0.04	0.18
Abundance	1	0.02	0.17	0.83	< 0.001
r^2^	85	0.91	< 0.001	0.94	< 0.001

Number of records (abundance) served as metric covariate.

Habitat conditions quantified by tree and shrub cover did not significantly influence abundances, species richness and community composition of the transects, except for a marginally significant positive correlation of tree cover and species richness. Similarly, butterfly ecological traits did not significantly co-vary with habitat conditions (see Appendix S1).

With respect to our second research question, we found a higher species turnover (β-diversity) of the transects located in the Kainaini river system than at Nzeeu during the rainy season; this difference was not apparent during the dry season (Table 1, Fig. 2c). Permanova confirmed structural differences between both sites and seasons, while pointing only to marginal differences with respect to distance from the rivers (Table 3).

**Table 3:** Two-way Permanova identified differences in butterfly community composition dependent on study site and season.

Variable	Partial η^2^	*P*
Study site	0.36	< 0.001
Season	0.16	< 0.001
Study site × season	0.12	< 0.001

Variable	Partial η^2^	*P*
Distance	0.04	0.03
Season	0.31	< 0.001
Distance × season	0.044	0.01

**Figure 3:**
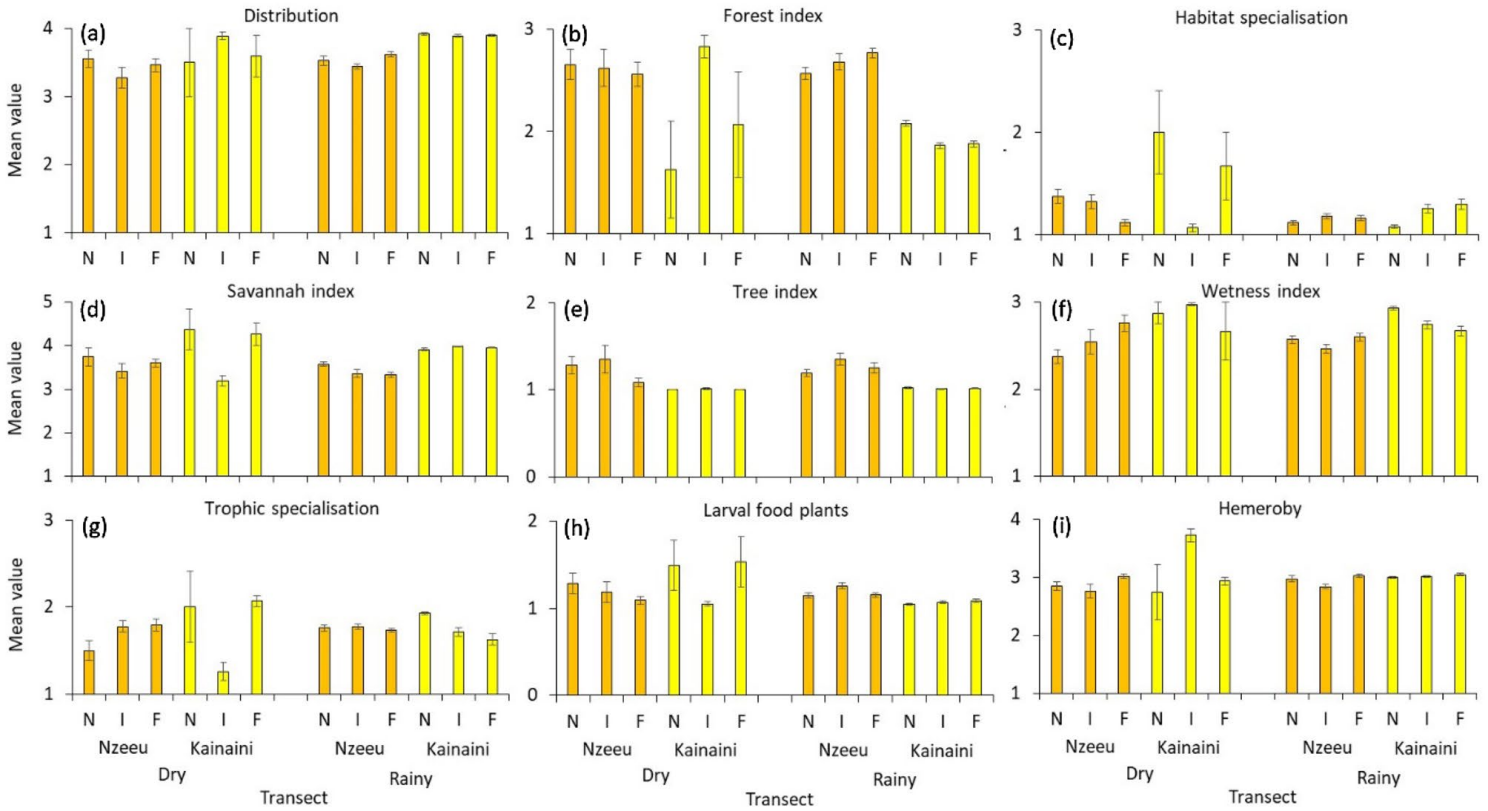
Average abundance-based butterfly ecological traits in transects along the Nzeeu (orange) and Kainaini (yellow) rivers near Kitui city in southern Kenia. Error bars denote standard errors. Traits used are (**a**) geographic distribution (4 categories), (**b**) forest index (5 categories), (**c**) habitat specialisation (3 categories), (**d**) savannah index (5 categories), (**e**) tree index (3 categories), (**f**) wetness index (3 categories), (**g**) larval foodplant specialisation (3 categories), (**h**) larval food plant type (dicotyledonous, monocotyledonous), and (**i**) hemeroby index (4 categories). Categories (apart from larval food plant type) are always in increasing order. Abbreviations: N—near river transects; I—transects at intermediate distance from river (i.e. 250 m); F—transects far from river (i.e. 500 m).

These differences in community compositions were not reflected by clear morphological differences between transect groups (Appendix S1), except for the forewing length/thorax width index that was consistently higher during the rainy season (Appendix S1), but we found significant differences in trait expression (Table 4, Fig. 3). Thus, based on mean values considering the number of individuals per species, habitat specialisation and savannah index differed significantly between the dry and the rainy season (Table 4, Fig. 3), indicating respective shifts in community composition of the adult individuals. The transects located in the Kainaini river system were comparatively richer in species occurring in open landscapes than at Nzeeu. Thus, the savannah index (Fig. 3d) was comparatively higher, the forest (Fig. 3b), tree (Fig. 3e) and wetness indices (Fig. 3f) lower at Kainaini River, indicating higher proportions of savannah species than in the Nzeeu site (P(F_1,94_) < 0.01). These differences were more pronounced for the butterflies on the wing during the rainy (P(F_1,94_) < 0.01) than during the dry season (P(F_1,94_) > 0.05). Seasons did not significantly differ with respect to the degree of hemeroby (Table 4, pairwise P(F_1,94_) > 0.05). Mostly similar values were obtained when these comparisons were based on means calculated from only presence-absence data (Appendix S1). However, contrary to the means obtained for numbers of individuals, the presence-absence data show a tendency toward higher values for habitat and larval food plant specialisation, underlining the presence of such specialist species, but in relatively low numbers.

**Table 4:** Differences identified with general linear modelling between transect distance from the river and season with respect to important butterfly habitat traits.

Variable	df	Habitat specialisation			Savannah index			Larval foodplant specialisation			Hemeroby index		
		β-value	Partial η^2^	*P*	β-value	Partial η^2^	*P*	β-value	Partial η^2^	*P*	β-value	Partial η^2^	*P*
Shrub cover	1	0.12	0.02	0.22	0.19	0.05	0.04	0.07	0.01	0.47	0.08	0.01	0.41
Tree cover	1	0.14	0.02	0.19	0.11	0.01	0.31	0.04	< 0.01	0.72	< 0.01	< 0.01	0.99
Abundance	1	0.07	< 0.01	0.64	− 0.12	0.01	0.40	− 0.04	< 0.01	0.80	0.06	< 0.01	0.72
Distance to river	2		0.06	0.07		0.13	< 0.001		0.08	0.03		0.07	0.05
Season	1		0.09	< 0.001		0.10	< 0.001		0.05	0.04		0.03	0.09
Distance to river × season	2		0.07	0.03		0.11	0.01		0.09	0.02		0.10	0.01
r^2^	87		0.31	< 0.001		0.32	< 0.001		0.23	< 0.01		0.24	< 0.01

Note that habitat conditions only marginally influenced trait expression. Number of records (abundance) served as metric covariate.

Answering our third research question, we found species composition to differ only marginally between the transect groups with different distances from the rivers, indicating considerable compositional similarity between transects near and far from the river (Tables 2, 3, Fig. 2d). At Nzeeu, but not at Kainaini, the respective compositional similarity of butterfly imagoes was higher during the rainy than during the dry season (Fig. 2d). Furthermore, average similarity across the three distance classes was even higher than between the transects near the river (Fig. 2d). We found the lowest compositional similarity (i.e. highest species turnover) within each transect group (Fig. 2d). There were no clear linear gradients correlated with distance from the river. The only case of a linear decrease was observed for the wetness index at the Nzeeu site during the dry season decreasing with increasing distance from the river (Fig. 3f).

## Discussion

### Species richness and abundance

We found a generally higher butterfly species richness and higher abundances (the latter only during the dry season) across the anthropogenic landscape disturbed along Nzeeu River if compared with the mostly undisturbed natural dry savannahs along Kainaini River. In contrast, abundances were higher in the mostly undisturbed study site during the rainy season. This picture is mostly congruent with results from other studies, showing higher species richness and abundances in diverse anthropogenic landscapes compared to natural habitats^7,16–19^. This also holds true for tropical ecosystems of East Africa where, for example in western Kenya, a higher bird species richness was found across heterogeneous agricultural land than in the adjoining natural forest patches^20^. Similarly, diversely structured urban areas with gardens may provide an even higher species richness and higher abundances than adjoining natural habitats do^21,22^. This result might be due to the accumulation of ecological niches and resources frequently found in anthropogenic landscapes. Particularly in dryland areas, such as in East Africa, artificial water irrigation produces more constant resource availability throughout the year, which boosts species richness and abundance^23,24^. In consequence, only specialist species adapted to survive rather dry climatic conditions were found along the near-natural Kainaini River site during the dry season and were much less frequent than in the anthropogenic landscape with higher artificial water availability.

Our transects along Nzeeu River represent a disturbed and heterogeneous environment consisting of a mosaic of gardens around habitations, fallow land, small fields for subsistence agriculture, trees and shrubs (the latter dominated by the exotic and persistently flowering shrub *Lantana camara*), as well as disturbed riparian forest remnants. This complex habitat diversity combined with year-round water irrigation of some parts of it provides numerous niches and a plenitude of resources for many butterfly species. However, the total number of individuals during the rainy season was higher in our savannah transects than in the agricultural land. This underlines the high potential of such near-natural sites to produce rather high numbers of individuals and the capacity for massive abundance of single species (e.g. Larsen^25^), as for *Belenois aurota* in our study.

### Community structure and specialist species

Apart from species richness and abundances, a closer look at the species composition is important in evaluating the ecological performance and conservation value of the two types of study sites. While, at a first glance, there is a positive effect on numbers of species and individuals in anthropogenically disturbed habitats, there is a lack of species with specific habitat requirements (e.g. several blues of the genera *Euchrysops* and *Lepidochrysops*, or the nymphalid *Pardopsis punctatissima*). However, these species were generally found at low abundances, also in the near-natural savannah habitats. Furthermore, several typical savannah species occurred at reduced levels of abundance in the study site dominated by human impacts (e.g. *Pinacopteryx eriphia*, *Charaxes xoolina*, *Junonia hierta*). This agrees with previous studies underlining that anthropogenic landscapes, although frequently richer in species numbers and abundances, do not hold specialist species^26^. Thus, rare bee species vanished after human disturbances of habitats in eastern North America^27^, and, in a study in the Taita Hills of southern Kenya, butterflies of cloud forests are mainly observable within the natural forest^28^. Thus, considering only the total number of species and individuals might lead to wrong interpretations of data. Consequently, a detailed look at the community structures and trait assemblages is necessary.

Our data show that community structures differed significantly between our two study sites. Differences in community assembly were particularly pronounced during the dry season, but less during the rainy season. The butterfly community in the anthropogenic landscape along Nzeeu River is comparatively diverse and consequently was expected to also span a broad trait space. In contrast, the butterflies found in the near-natural habitats along Kainaini River represent a partly distinct community consisting more of savannah elements, but reached almost the species richness found along Nzeeu River during the rainy season. However, the butterfly community in the anthropogenic landscape is more dominated by ubiquists, exemplified by considerably higher numbers of species such as *Catopsilia florella* and *Hypolimnas misippus*. Here we also found common species like *Neptis saclava* and *Bicyclus safitza* which require more dense vegetation (as provided by the garden and farmland structures) but have few additional habitat requirements. In contrast, typical species of dry savannah habitats (such as *Pinacopteryx eriphia*, *Charaxes xoolina* and *Junonia hierta*) are considerably more frequent in the largely undisturbed savannah environment along Kainaini River. Thus, our data reflect the typical pattern that community structures follow environmental conditions and thus mirror human disturbances, as also demonstrated for other tropical ecosystems^28,29^, and that generalist species frequently replace specialist species after human disturbances^28,30^. In summary, anthropogenic landscapes may host a high number of species and individuals, but lack the specialist species that respond highly sensitively to habitat disturbances.

### Seasonal shifts

Seasonal shifts in tropical ecosystems significantly impact the prevailing environmental conditions, because the availability of resources strongly depends on the amount of precipitation^31^. Insect populations are known to react very strongly to these seasonal effects, particularly in the dryland ecosystems of East Africa^2^. However, the detectable effect of seasonality in our study was less pronounced in the anthropogenic landscape (5131 counted butterfly imagoes during the rainy season, 459 individuals during the dry season) than in the near-natural savannah site (8081 vs. 82). Obviously, the seasonal fluctuation in the number of adult individuals is buffered in anthropogenic habitats by multiple factors: 1. Permanent water irrigation ensures resource availability throughout the year, and, 2. Species at home in anthropogenic landscapes often have broader ecological amplitudes, and their response might thus be more plastic than that of specialist species. Previous studies also showed similar seasonal patterns in the number of adult individuals and differences in community structures, depending on landscape configuration^20^. Thus, seasonal shifts of adult butterfly community composition and abundance in the East African coastal forest ecosystem is also buffered in anthropogenic landscapes (e.g. agriculture, forest edges and tree plantations), but are very pronounced in the natural dryland forest habitat^2^. Consequently, the here observed seasonality pattern of communities represents a general feature in butterflies (and insects more generally) of the drylands of tropical Africa.

### Spill-over effects

Answering our third research question, we found no significant differences in species richness, abundance and community structure between transects in the riparian forest on the one hand compared to the transects in the dry savannah and the agricultural land on the other. Furthermore, our data do not show any significant gradient of butterfly diversity spill-over from the riparian forests into the adjoining landscapes. Our findings contrast with other studies indicating positive spill-over effects from (near) natural habitats into anthropogenic landscapes^32^. Such spill-over effects might have a positive influence on ecosystem functions (i.e. services) such as an increase in pollination due to visiting pollinators from (near) natural habitats^33^, which might enhance agricultural productivity^32,34–36^. In our study, however, the size of the gallery forests seems to be too small, and they are too closely intertwined with the adjacent ecosystems, so that potential spill-over effects are not detectable.

## Methods

### Study sites

Our study sites are located on the Yatta Plateau in south-eastern Kenya. This region is characterized by dry savannahs. Annual rainfall (average: 810 mm) occurs during two periods, from March to May (average: 330 mm) and from October to January (average 480 mm) (c.f. Jaetzold et al.^37^). The commonest soil types are ferralsols and luvisols, which are of low fertility^37^. 97.1% of the human population in our study region depend on subsistence crop farming^38^, and the population has almost doubled in number from 1999 to 2009^38^. Consequently, fallow periods for fields are omitted, which further decreases soil fertility, and increases pressure on pristine habitats.

The dry savannah landscape is traversed by temporary (seasonal) rivers. These rivers are bordered by riparian vegetation, consisting of a diverse and unique plant community. However, this vegetation is frequently exploited for timber, charcoal and brick production^39,40^. The region is further affected by climate change, with an increase in rainfall variability and mean temperature^37^. These factors lower the reliability of agricultural production and food security, hence leading to severe destruction of pristine habitats.

We selected two study sites, affected by different anthropogenic pressures, but which are subject to identical biotic and abiotic preconditions (including seasonality): Firstly, a highly degraded anthropogenic landscape along Nzeeu River, south of Kitui city. Secondly, a largely intact dryland environment along Kainaini River located near the university campus of the South Eastern Kenya University, north of Kitui city (Fig. 1). The landscape along Nzeeu River is densely populated by subsistence farmers. Thus, the original riparian and savannah vegetation has been mostly transformed into arable fields for the cultivation of maize, sorghum, peas, and mangos. Furthermore, the riparian vegetation, where it still exists, has largely been replaced by invasive exotic plant species (e.g. *Lantana camara*)^12^. The landscape of our second study site along Kainaini River represents a still largely intact riparian forest with adjoining dry savannahs. It remains mostly undisturbed, except for some moderate live-stock pasturing by nearby subsistence settlers.

### Butterfly assessments

We counted butterflies in both habitat types along line-transects, each 150 m long. We set 24 transects along each of the two rivers, with eight transects along the river bank, eight 250 m distant to the river, and another eight 500 m distant to the river (in total: 2 × 24 transects = 48 transects). The minimum distance between transects was at least 200 m, to minimize spatial autocorrelation. Exact GPS coordinates of each transect are given in Appendix S2.

We recorded all butterflies encountered during transect counts (species, number of individuals of each species). Each transect was visited eight times during the dry season (August/September 2019) and eight times during the rainy season (January/February 2020). Data collection was performed between 9 a.m. and 4 p.m. Each butterfly individual within 5 m of the transect line (horizontally to vertically) was recorded by visual observation and, if needed, a butterfly net (see Pollard^15^, with modifications). While recording butterflies, the observers walked very slowly and spent about 15 min per transect. Species were identified either immediately while the butterfly was on the wing, or individuals were netted and then determined in the field. Individuals of species for which ad hoc identification was critical (e.g. many blues and skippers) were caught with the net, photographed (upper and under wing side) and released again. The photograph-based identification of these individuals was performed later using literature^25^. Apart from species and number of individuals per species, we recorded cloud cover during each transect walk (classified as: clear, slightly cloudy, mostly cloudy, overcast), exact time, and date. Field teams comprised two observers and one person making notes of all observations. Transects are displayed in Fig. 1. All butterfly data collected are compiled in Appendix S3.

### Traits

The occurrence of a species in a specific environment strongly depends on its ecology, behaviour, and life-history^41^. Therefore, we considered these characteristics for each butterfly species recorded in the field. These trait data were compiled from Larsen^25^ and web-sites (e.g. www.gbif.org, www.lepiforum.de/non-eu.pl). We considered the following characteristics: wing span (mm), ratio length/width of the forewing (relative), ratio forewing length/thorax width (relative), geographic distribution (4 categories), savannah index (5 categories), forest index (5 categories), tree index (3 categories), wetness index (3 categories), habitat specialisation (3 categories), larval foodplant specialisation (3 categories), larval food plant type (dicotyledonous, monocotyledonous), and hemeroby index (4 categories). Detailed classifications are provided in Appendix S4.

### Habitat parameters

Habitat structures impact species´ occurrence, abundances and community structures^42^. In our study, we considered habitat structures for each transect. Habitat parameters were recorded (counted and estimated) every 20 m along each transect. We estimated the following habitat parameters: Canopy cover (percentage of leaf cover vs. sky measured with the CanopeoApp); herb, shrub and tree cover (percentage coverage of each layer within a radius of 3 m); flowers on herbs, shrubs and trees (estimated within a radius of 3 m, and subsequently allocated to the classes 0, 1–10, 11–50, 51–100 and > 100 flowers); occurrence of *Lantana camara* shrub, and exotic trees (estimated coverage within a radius of 3 m, and subsequently allocated to the classes 0 (no), 1 (rare), 2 (present) and 3 (dominant), respectively); and water availability (presence/absence) within a radius of 3 m. All raw data of habitat parameters are provided in Appendix S5.

### Statistics

We first arranged the raw data in three matrices: a 71 × 14 species × trait matrix **T**, a 71 × 96 species × transect matrix **M**, and a 6 × 96 habitat characteristics × transect matrix **H**. Matrix multiplication of **E** = **T**^−1^**MA**^−**1**^, where **A** is the vector of total abundances in the transects, returned a matrix **E** of average trait expression in each transect.

To answer the first research question, we compared species richness, abundances, and trait expression between the transects and used general linear modelling (glm) to detect differences in richness and trait expression with respect to the study sites (i.e. the two river systems with their different land-use patterns), season, distance from the rivers, as well as to environmental variables. Some of the habitat variables and trait expressions were highly positively correlated (Appendix S1). Consequently, the glm included only variables correlated by less than r = 0.7 (i.e. shrub cover, tree cover, habitat specialisation, savannah index, larval foodplant specialisation, and hemeroby).

To infer differences in community structure between transects (second research question), we first calculated the two most dominant eigenvectors, which explained 91.5% and 3.5% of variance, of a principal components analysis of the **M** matrix. These eigenvectors cover differences in species composition between and within transects. We used glm and two-way Permanova to relate these differences to season, distance to river, and study sites (i.e. different land-use types in the two river systems). Additionally, we assessed the degree of β-diversity among sets of transects with the proportional turnover metric of Tuomisto^43^: $\beta =1-\frac{\alpha }{\gamma }$; where α denotes the average species richness per transect and γ the corresponding total richness.

To infer species spill-over effects from the riparian forests into the adjoining savannah (third research question), we calculated the Bray–Curtis similarities for three groups of transects within each season and study site. First, we compared average pairwise Bray–Curtis values between transects of intermediate and greater distance with the near-river transects within each study site. Second, we calculated the average Bray–Curtis similarities between all transects within each study site (2)—season (2)—distance class to river (3) combination. Third, we calculated the average within-transect Bray–Curtis similarity for the rainy season, to infer small scale compositional variability. The latter calculations were impossible for the dry season, due to the overall low number of recorded species. Calculations were done with Statistica 12.

## Supplementary Information

Supplementary Information 1.

Supplementary Information 2.

Supplementary Information 3.

Supplementary Information 4.

Supplementary Information 5.

Supplementary Information 6.

Supplementary Information 7.
